# Adjusting for One Issue while Ignoring Others Can Make Things Worse

**DOI:** 10.1371/journal.pone.0120817

**Published:** 2015-03-18

**Authors:** Alan H. Welsh, David B. Lindenmayer, Christine F. Donnelly

**Affiliations:** Australian National University, Canberra, Australia; Utah State University, UNITED STATES

In [1], we presented an example to show that using occupancy modelling to adjust for imperfect detection can make the bias worse than not making any adjustment for imperfect detection. The intuition behind the example is that imperfect detection is only one of a number of problems that can introduce measurement error into the data and it is a mistake to focus exclusively on only one kind of measurement error and ignore other kinds. Our example can be regarded as a counter example against the widespread belief that occupancy modelling is a universally applicable approach for handling imperfect detection. As is usual with counter examples, the example was constructed to make a strong point, but it was inspired by a real empirical study using data gathered from 55 long-term sites surveyed repeated in 8 years between 1998 and 2009. This dataset provided insight into the kind of counter example that would make the adjustment from occupancy modelling misleading.

Guillera-Arroita et al have presented in [2] a counter counter example to show that occupancy modelling sometimes works and, on this basis, argue that it is dangerous not to adjust for non-detection. Counter counter examples have no standing in logic and do not refute counter examples. Indeed, [1] also contained ideal examples where detection/occupancy modelling works. Specifically, we presented examples in which imperfect detection was the only source of measurement error. A key point made in [1] is that we cannot tell empirically from the data whether we have a case in which imperfect detection is the only source of measurement error or not. This means that we cannot tell whether adjustment for imperfect detection improves the estimates, has no effect or makes the estimates worse. We can claim as a matter of belief (that other scientists are free to reject) that imperfect detection is the only source of measurement error, but we cannot demonstrate this empirically. Moreover, variation in abundance and the use of detection methods that are affected by abundance means that we are often (and indeed usually) in a situation where imperfect detection is not the only source of measurement error. As shown in [1], in such cases, the adjustment can be harmful by an unknown but possibly large amount.

We agree that we should not simply ignore imperfect detection. However, as we demonstrated in [1], standard adjustments can often make things worse and we cannot tell empirically whether adjustment is improving or making the situation worse. This makes imperfect detection a difficult problem that the currently available methods do not solve and means that the application of these methods in all cases is not justified.
